# Insulin increases sensory nerve density and reflex bronchoconstriction in obese mice

**DOI:** 10.1172/jci.insight.161898

**Published:** 2022-10-24

**Authors:** Gina N. Calco, Jessica N. Maung, David B. Jacoby, Allison D. Fryer, Zhenying Nie

**Affiliations:** Division of Pulmonary and Critical Care Medicine, Oregon Health & Science University, Portland, Oregon, USA.

**Keywords:** Metabolism, Pulmonology, Asthma

## Abstract

Obesity-induced asthma responds poorly to all current pharmacological interventions, including steroids, suggesting that classic, eosinophilic inflammation is not a mechanism. Since insulin resistance and hyperinsulinemia are common in obese individuals and associated with increased risk of asthma, we used diet-induced obese mice to study how insulin induces airway hyperreactivity. Inhaled 5-HT or methacholine induced dose-dependent bronchoconstriction that was significantly potentiated in obese mice. Cutting the vagus nerves eliminated bronchoconstriction in both obese and nonobese animals, indicating that it was mediated by a neural reflex. There was significantly greater density of airway sensory nerves in obese compared with nonobese mice. Deleting insulin receptors on sensory nerves prevented the increase in sensory nerve density and prevented airway hyperreactivity in obese mice with hyperinsulinemia. Our data demonstrate that high levels of insulin drives obesity-induced airway hyperreactivity by increasing sensory innervation of the airways. Therefore, pharmacological interventions to control metabolic syndrome and limit reflex-mediated bronchoconstriction may be a more effective approach to reduce asthma exacerbations in obese and patients with asthma.

## Introduction

There are over 250,000 new obesity-related asthma cases per year in the United States (1–6). Over 60% of the adults with severe asthma are obese (7, 8) and respond poorly to typical asthma medications, such as corticosteroids, leading to higher healthcare costs (9) and a substantially reduced quality of life (6, 10). However, the mechanisms driving obesity-related asthma are unclear, limiting our ability to develop new treatments.

Dysfunctional airway nerves lead to airway hyperreactivity, the excessive bronchoconstriction response to stimuli that is a defining feature of asthma. In the airways, sensory neurons in the epithelium detect stimuli in the lumen and relay this signal through the central nervous system. This relay activates parasympathetic nerves, the dominant autonomic nerves controlling airway smooth muscle (11). Parasympathetic nerves release acetylcholine, which activates M_3_ muscarinic receptors on airway smooth muscle, causing bronchoconstriction. Release of acetylcholine is limited by inhibitory M_2_ muscarinic receptors on prejunctional parasympathetic nerves. Loss of neuronal M_2_ muscarinic receptor function is a well-known mechanism of airway hyperreactivity that has been demonstrated in humans, allergic asthma, and in every animal model of asthma studied (12), including in rats with obesity-induced asthma (13, 14). Activation of sensory nerves and subsequent reflex bronchoconstriction is also significantly increased in patients with asthma (15, 16), also showing marked, abnormal structural changes (17). However, the architecture and function of airway sensory nerves have never been investigated in obese individuals. Here, we studied the contribution of sensory nerves to increased activation of neural reflexes in obesity-related asthma.

Insulin resistance and compensatory hyperinsulinemia are common in obese individuals and increase the risk of asthma independently of other variables (18, 19). Disruption of insulin signaling by obesity has been linked with airway hyperreactivity and asthma (20). Insulin potentiates parasympathetic, nerve-mediated bronchoconstriction in obese rats by impairing M_2_ muscarinic function (13). At physiological levels, insulin also enhances sensory neurite outgrowth and acts directly on sensory nerves to promote axon growth and regeneration (21). Thus, hyperinsulinemia may increase nerve-mediated reflex bronchoconstriction by affecting the structure or function of sensory nerves and parasympathetic nerves, as both are components of the reflex bronchoconstriction pathway.

In this study, we tested the effects of insulin on airway nerve–mediated reflex bronchoconstriction in a diet-induced obese mouse model, which has increased circulating insulin. Our data demonstrate that airway epithelial sensory innervation and airway nerve–mediated reflex bronchoconstriction were increased in hyperinsulinemic obese mice. Selectively depleting insulin receptors on sensory nerves of hyperinsulinemic, obese mice prevented this increase in sensory nerve density.

## Results

### Mice on a high-fat diet were obese, hyperinsulinemic, and hyperglycemic.

**Figure 1:**
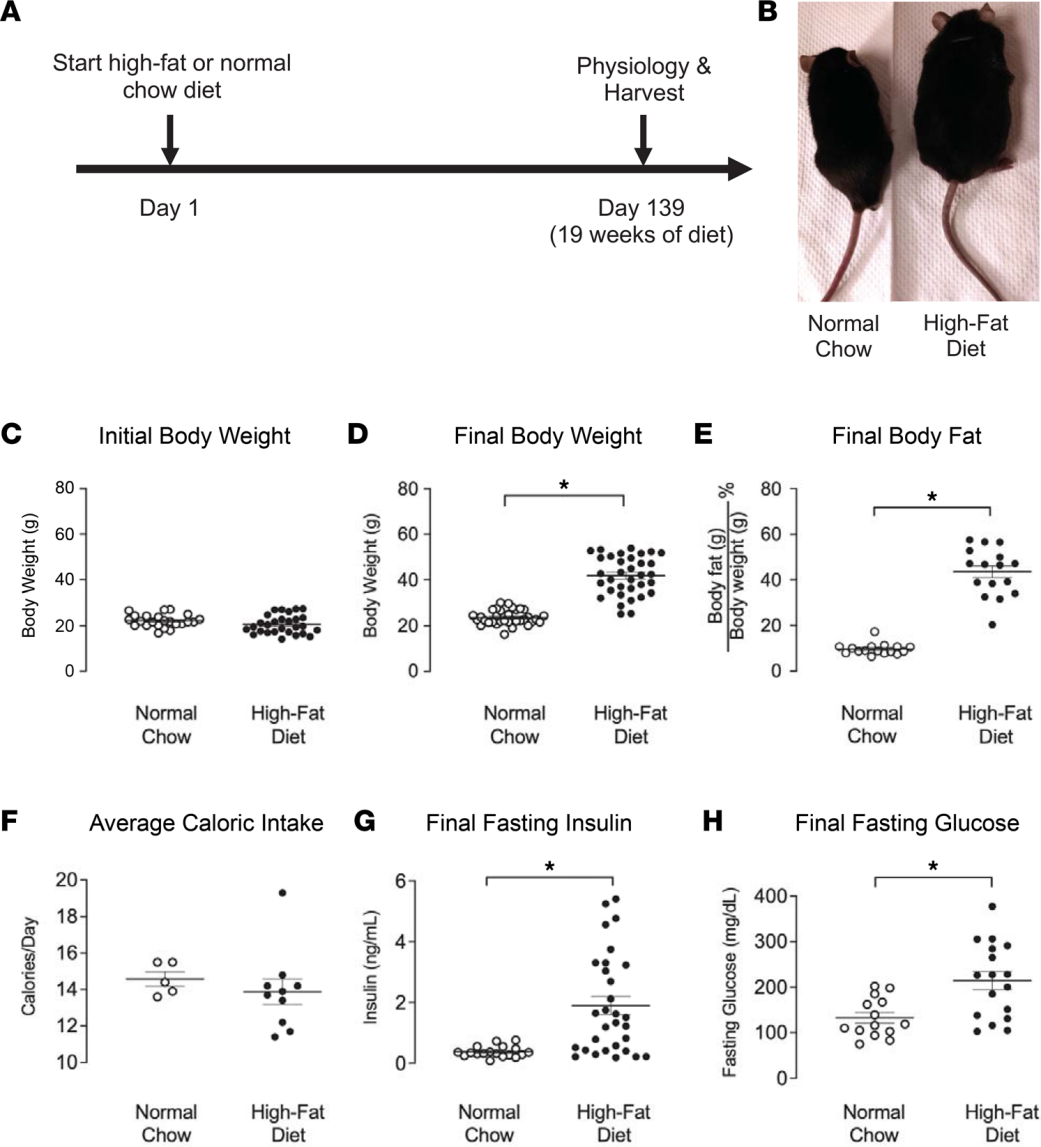
High-fat diet–fed mice were obese, hyperinsulinemic, and hyperglycemic. (**A**) WT mice were started on either high-fat diet (HFD) or normal chow diet for 19 weeks. (**B**) Representative pictures of normal chow–fed and high-fat diet–fed mice after 19 weeks of diet. (**C**) Both animal groups had similar initial body weights. (**D** and **E**) WT high-fat diet–fed mice (closed circles) had a significant increase in postdiet body weight (**D**) and percent body fat (**E**) compared with WT normal chow–fed mice (open circles). (**F**) Caloric intake was similar among animals. (**G** and **H**) High-fat diet fed mice had significantly increased fasting insulin (**G**) and fasting glucose (**H**). In all graphs except **F**, each data point represents an individual animal (*n* = 14–23); however, in **F**, the weight of chow was measured by cage; thus, in **F**, each point represents a cage containing 2–5 animals. Data are represented as mean ± SEM, using 2-tailed Student’s *t* test. *P < 0.05.

Body weight in mice fed a high-fat diet increased by 77% compared with age-matched mice on a normal chow diet (Figure 1, B and D), although their body weights were similar before diet treatment (Figure 1C). Body fat also increased by 345% in mice on a high-fat diet compared with those on a normal chow diet (Figure 1E). Despite an increase in both body weight and fat in the high-fat diet group, mice on both diets ate a similar number of calories per day (Figure 1F). A high-fat diet significantly increased fasting insulin (2.27 ± 1.66 ng/mL compared with 0.40 ± 0.17 ng/mL in those on a normal chow diet) and fasting glucose (214 ± 82.4 mg/dL compared with 140 ± 42.5 mg/dL in those on a normal chow diet) (Figure 1, G and H). These data are consistent with previous reports (22) indicating that we have successfully established our diet-induced obese mouse model to measure the effects of neurally mediated airway reactivity.

### High-fat diet–fed mice had increased reflex bronchoconstriction.

**Figure 2:**
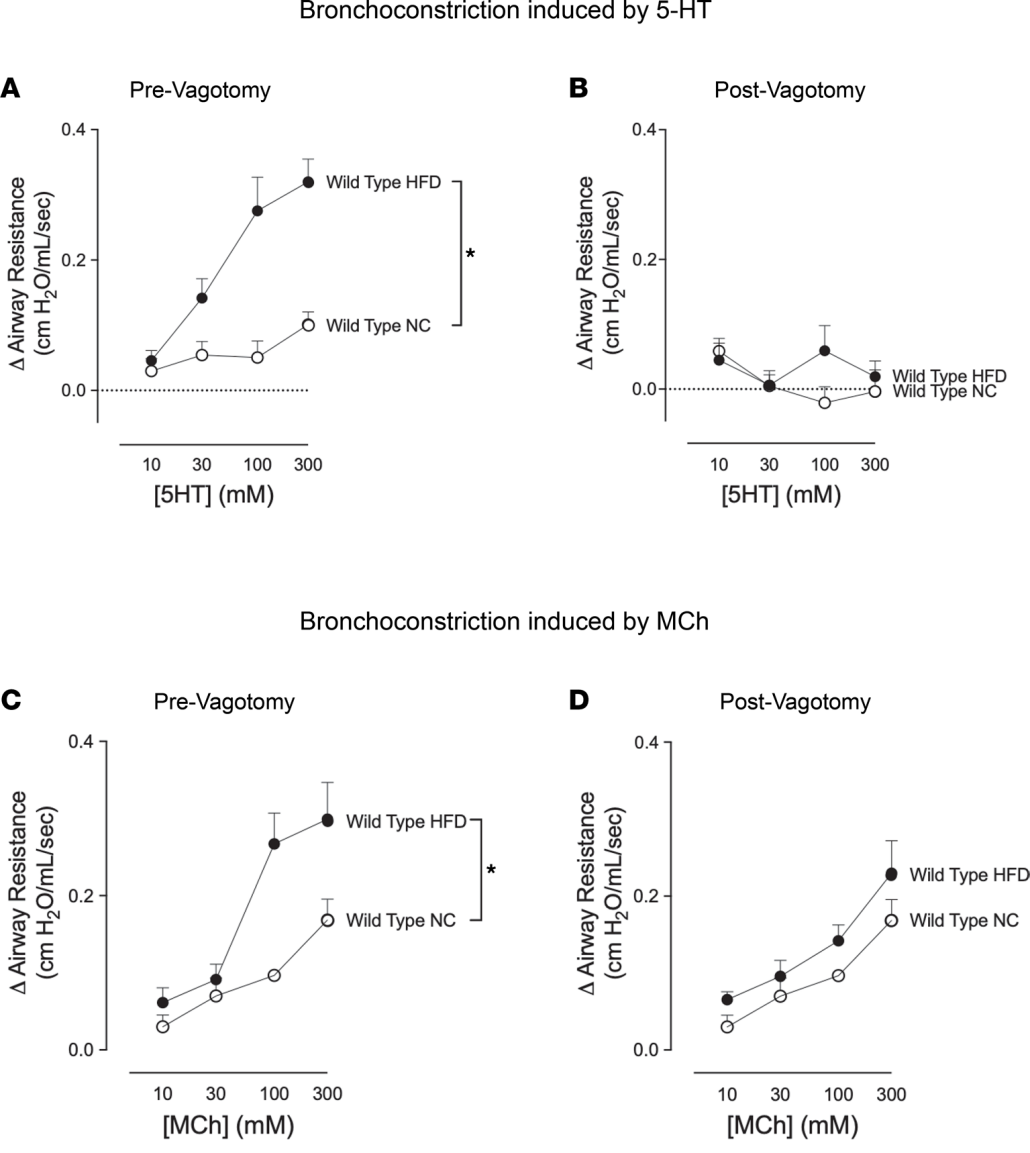
High-fat diet–fed (HFD-fed) mice had increased reflex bronchoconstriction. (**A**) Change in airway resistance was measured in anesthetized and mechanically ventilated mice. 5-HT induced dose-dependent bronchoconstriction that was significantly greater in mice fed HFD (filled circles) than in mice fed normal chow (open circles). (**C**) Mice on a HFD also had increased bronchoconstriction in response to inhaled methacholine (MCh) compared with normal chow–fed mice. (**B** and **D**) The increased bronchoconstriction in response to either 5-HT (**B**) or MCh (**D**) was blocked by vagotomy in mice on a HFD, indicating this increase was mediated by a reflex response. Each data point represents the mean ± SEM, using 2-way repeated-measures analysis of variance (*n* = 6–12). *P < 0.05.

5-hydroxtryptamine–induced (5-HT–induced) bronchoconstriction was significantly greater in mice fed a high-fat diet compared with mice fed normal chow (Figure 2A). This increased bronchoconstriction in response to 5-HT was blocked by vagotomy (Figure 2B) or by atropine (data not shown), indicating that the increased bronchoconstriction was due to a potentiated vagal reflex. Bronchoconstriction in response to inhaled methacholine (MCh), a M_3_ muscarinic receptor agonist, was significantly increased in mice with intact vagus nerves on a high-fat diet compared with normal chow (Figure 2C). This increased response to MCh was also eliminated by vagotomy, indicating that increased bronchoconstriction was due to a potentiated vagal reflex. In vagotomized mice, inhaled MCh acts directly on airway smooth muscle to cause contraction. Our data demonstrate that there was no difference in MCh-induced bronchoconstriction between vagotomized mice on a high-fat diet compared with those fed normal chow (Figure 2D).

### High-fat diet–fed mice did not have airway inflammation.

**Figure 3:**
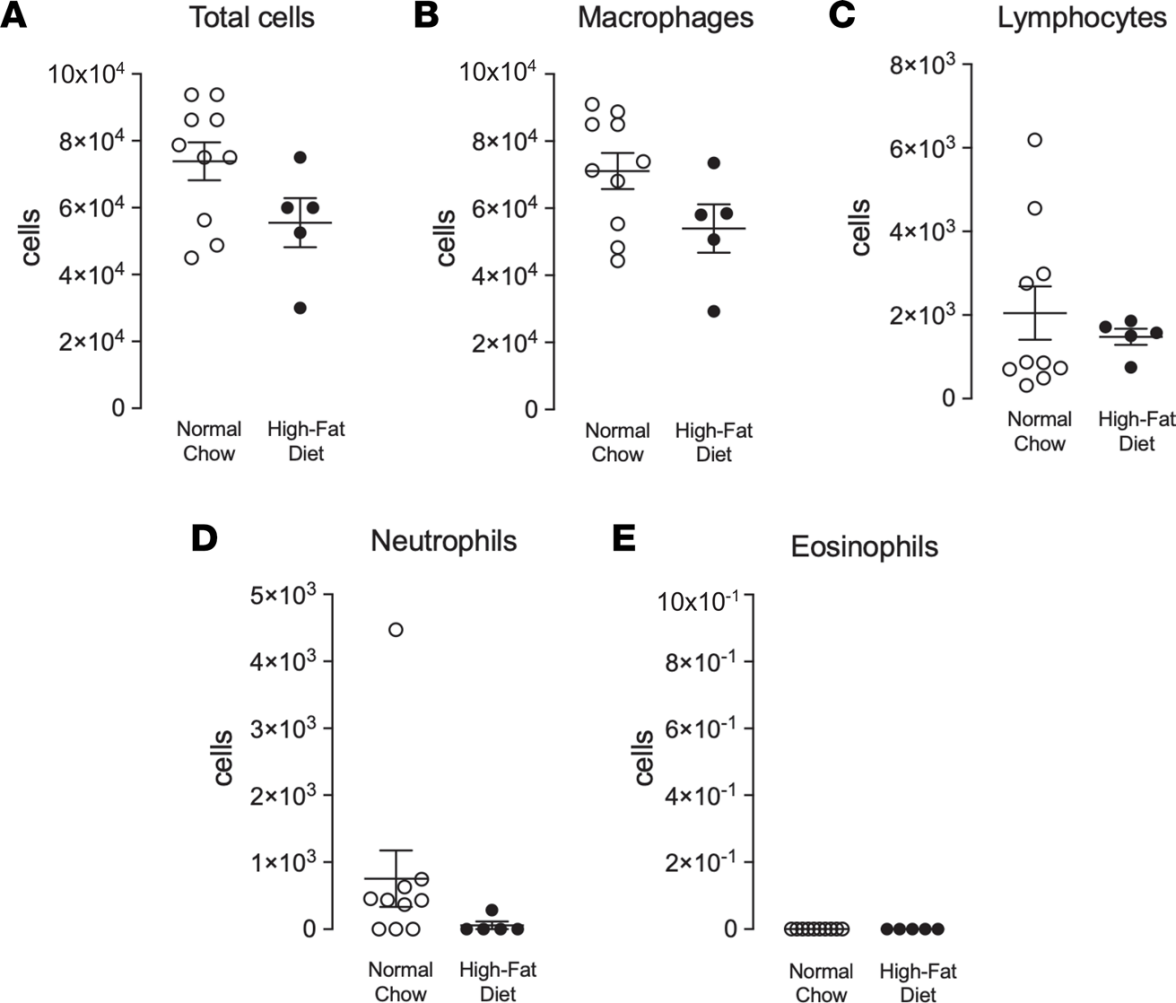
There is no evidence of cellular inflammation in lungs of mice on a high-fat diet. (**A**) Normal chow–fed (open circles) and high-fat diet–fed (filled circles) mice have similar total cell numbers in their bronchioalveolar lavage fluid. (**B**–**E**) There was no diet-induced difference in macrophages (**B**), lymphocytes (**C**), neutrophils (**D**), or eosinophils (**E**). Each data point represents an individual animal; data are represented as mean ± SEM using 2-tailed Student’s *t* test (*n* = 5–10).

Both normal chow and high-fat diet–fed mice had a similar total number of cells in their bronchoalveolar lavage fluid (BALF) (Figure 3A). Both diet groups also had similar numbers of macrophages, lymphocytes, neutrophils, and eosinophils (Figure 3, B–E). Neither group had any lung infiltration of inflammatory cells typically present in allergic asthma (23).

### Insulin receptor expression decreased on sensory neurons of SNIRKO mice treated with tamoxifen.

**Figure 4:**
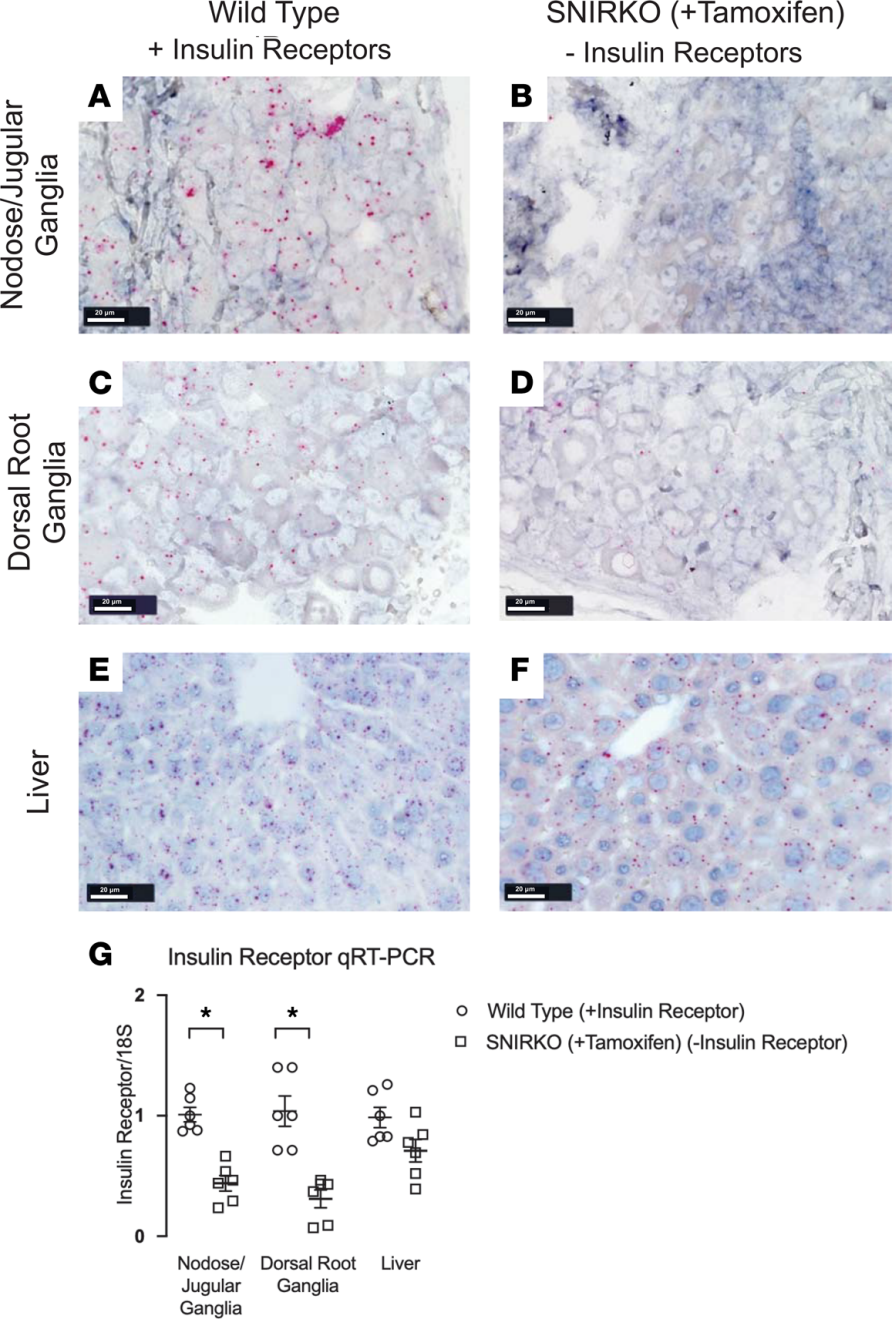
Creation and characterization of sensory neuron insulin receptor–KO (SNIRKO) mice. (**A–F**) Representative images of insulin receptor mRNA expression shown by Fast Red staining (pink dots) in WT mice with intact insulin receptors (IR^+^) and SNIRKO mice with selectively deleted insulin receptors (IR^–^). Tamoxifen treated SNIRKO mice (IR^–^) had decreased insulin receptor mRNA detected in the nodose and jugular ganglia (**B**) and dorsal root ganglia (**D**) compared with mice with intact insulin receptors in **A** and **C**. Liver of both mice groups had similar insulin receptor mRNA expression (**E** and **F**). (**G**) The relative changes in the amount of mRNA in sensory neurons and liver cells were measured by qPCR. Data are represented as mean ± SEM using 2-tailed Student’s *t* test (*n* = 6). Scale bars: 20 μm. *P < 0.05.

Our inducible sensory neuron insulin receptor–KO (SNIRKO) mice were generated differently than the mice used by Grote et al. (24). Thus, we first verified the depletion of insulin receptor mRNA expression in sensory neurons by the BaseScope in situ hybridization technique, which was used to locate mRNA expression at the single-cell level (Figure 4, A–F). mRNA expression was visualized via pink dots present in images in Figure 4, A–F. Insulin receptor mRNA quantification was confirmed by quantitative PCR (qPCR) in liver, dorsal root ganglia (DRG), and nodose and jugular ganglia (Figure 4G). After treatment with tamoxifen, insulin receptor mRNA expression in SNIRKO mice was significantly decreased in DRG compared with that found in WT mice (Figure 4, C, D, and G). Because both the nodose and jugular vagal ganglia provided sensory innervation to the airways, we further confirmed that insulin receptor mRNA expression was reduced in nodose and jugular ganglia of tamoxifen-treated SNIRKO mice (Figure 4, A, B, and G). In nonneuronal cells, such as liver cells, tamoxifen did not decrease mRNA expression, which demonstrated that insulin receptor KO was limited to sensory nerves (Figure 4, E–G).

### Selectively depleting insulin receptors on sensory neurons increased circulating insulin.

**Figure 5:**
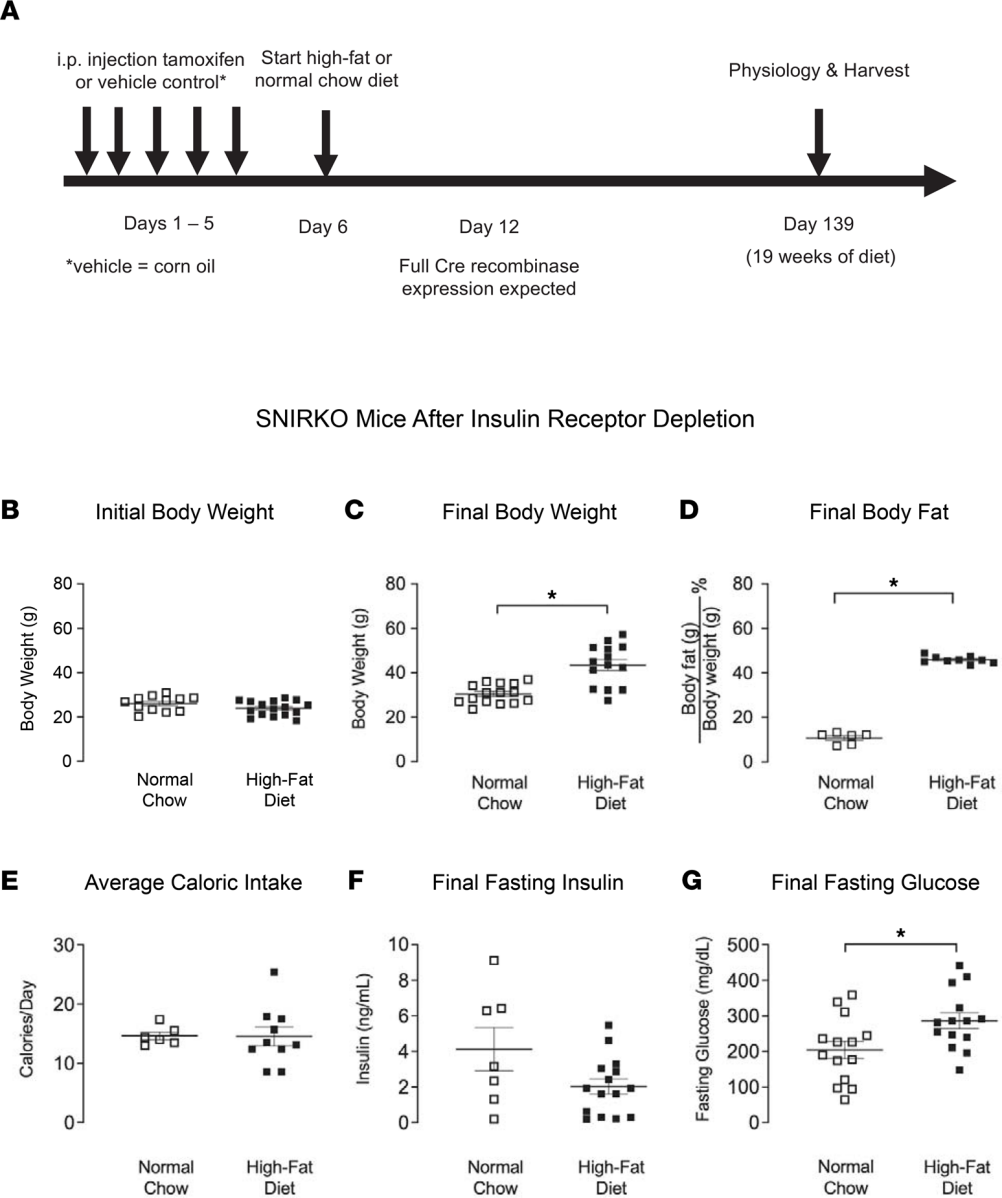
Characterization of SNIRKO mice treated with tamoxifen on normal chow or high-fat diet. (**A**) Male and female SNIRKO mice were treated with 5 consecutive days of i.p. tamoxifen (20 mg/mL) or corn oil (vehicle control) to induce Cre recombinase activity and delete the insulin receptors on sensory neurons. On day 6, they were started on either a high-fat diet or normal chow diet for 19 weeks. Full Cre recombinase expression in SNIRKO mice was expected by day 12. (**B**) SNIRKO mice had similar initial body weights before starting specific diets. (**C** and **D**) SNIRKO mice had significantly increased body weight (**C**) and body fat (**D**) after a high-fat diet. (**E**) Mice consumed a similar number of calories per day regardless of diet. (**F** and **G**) Both diet groups had similar fasting insulin (**F**), while high-fat fed diet mice had slightly but significantly higher glucose levels (**G**). Each data point represents an individual animal; data are represented as mean ± SEM using 2-tailed Student’s *t* test (*n* = 10–15). *P < 0.05.

Selectively depleting insulin receptors on sensory neurons significantly increased fasting insulin in SNIRKO mice compared with control mice (WT mice on a normal chow), regardless of diet (WT normal chow, 0.40 ± 0.17 ng/mL; SNIRKO normal chow, 4.12 ± 3.22 ng/mL [*P* < 0.0001]; SNIRKO HFD, 2.03 ± 1.63 ng/mL [*P* = 0.0314]). SNIRKO mice treated with tamoxifen on a high-fat diet had similar fasting insulin levels compared with normal chow diet–fed mice (Figure 5F), and both were higher than those in WT mice on a normal chow diet (Figure 1G and Supplemental Figure 1; https://doi.org/10.1172/jci.insight.161898DS1). This indicated that selectively depleting insulin receptors on sensory nerves caused hyperinsulinemia in comparison with normal chow–fed WT mice. High-fat diet–fed SNIRKO mice also had slightly, but significantly, higher fasting glucose levels compared with SNIRKO mice on a normal chow diet (286 ± 83.0 mg/dL on high-fat diet compared with 204 ± 91.5 mg/dL on normal chow diet) (Figure 5G). All mice had similar body weights when the diet treatment started (Figure 5B). However, mice with insulin receptors selectively depleted on sensory neurons gained more body weight and body fat on a high-fat diet compared with those on a normal chow diet (Figure 5, C and D). Daily caloric intake was similar in both diet groups (Figure 5E).

### High-fat diet–induced potentiation of reflex bronchoconstriction is prevented by selectively depleting insulin receptors on sensory nerves (using SNIRKO mice).

**Figure 6:**
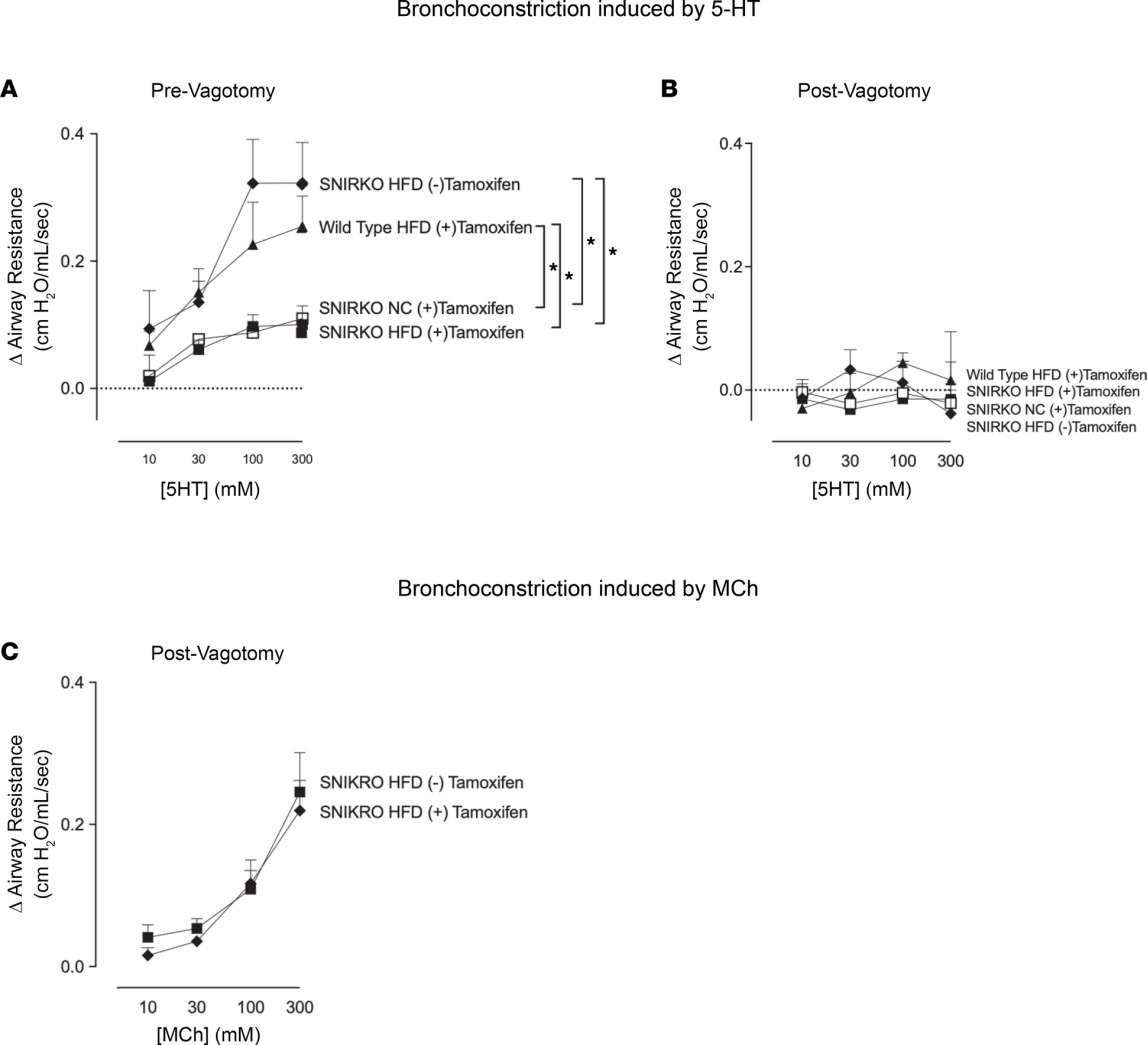
Specific KO of insulin receptor in sensory neurons prevented potentiated reflex bronchoconstriction in mice on a high-fat diet. (**A**) Airway resistance was measured in anesthetized and mechanically ventilated sensory insulin receptor–KO (SNIRKO) mice. 5-HT induced dose dependent bronchoconstriction in high-fat diet–fed SNIRKO mice without tamoxifen treatment (filled diamonds). Knocking out insulin receptors on sensory nerves with tamoxifen significantly inhibited 5-HT induced bronchoconstriction resulting from a high fat diet (filled squares). Tamoxifen alone did not inhibit 5-HT induced bronchoconstriction in WT mice on a high fat diet (filled triangles) or inhibit 5-HT induced bronchoconstriction in SNIRKO mice on a normal diet (open squares). (**B**) Response to 5-HT was eliminated by vagotomy in all mice. (**C**) Following vagotomy, bronchoconstriction in response to inhaled methacholine (MCh) was similar in SNIRKO mice with and without tamoxifen on a high-fat diet. Each data point represents the mean ± SEM using 2-way repeated-measures ANOVA (*n* = 6–7). *P < 0.05.

When fed a high-fat diet, SNIRKO mice without tamoxifen treatment (i.e., with intact insulin receptors on sensory nerves) had increased reflex bronchoconstriction to inhaled 5-HT, similar to that seen in the WT high-fat diet–fed mice without tamoxifen (Figure 6A). This increased bronchoconstriction in response to 5-HT was blocked by vagotomy (Figure 6B), indicating that the response to 5-HT was neurally mediated. WT animals on a high-fat diet with and without tamoxifen had similarly increased reflex bronchoconstriction, demonstrating that tamoxifen alone did not inhibit 5-HT induced bronchoconstriction. In contrast, tamoxifen in SNIRKO mice effectively depleted insulin receptors on sensory nerves and completely prevented the increased reflex bronchoconstriction to inhaled 5-HT in mice on a high-fat diet. Vagotomized SNIRKO mice with and without tamoxifen had similar bronchoconstriction responses to MCh (Figure 6C). Therefore, reduced bronchoconstriction in SNIRKO mice treated with tamoxifen was not due to changes in smooth muscle function.

### High-fat diet–fed mice have increased airway epithelial sensory innervation.

**Figure 7:**
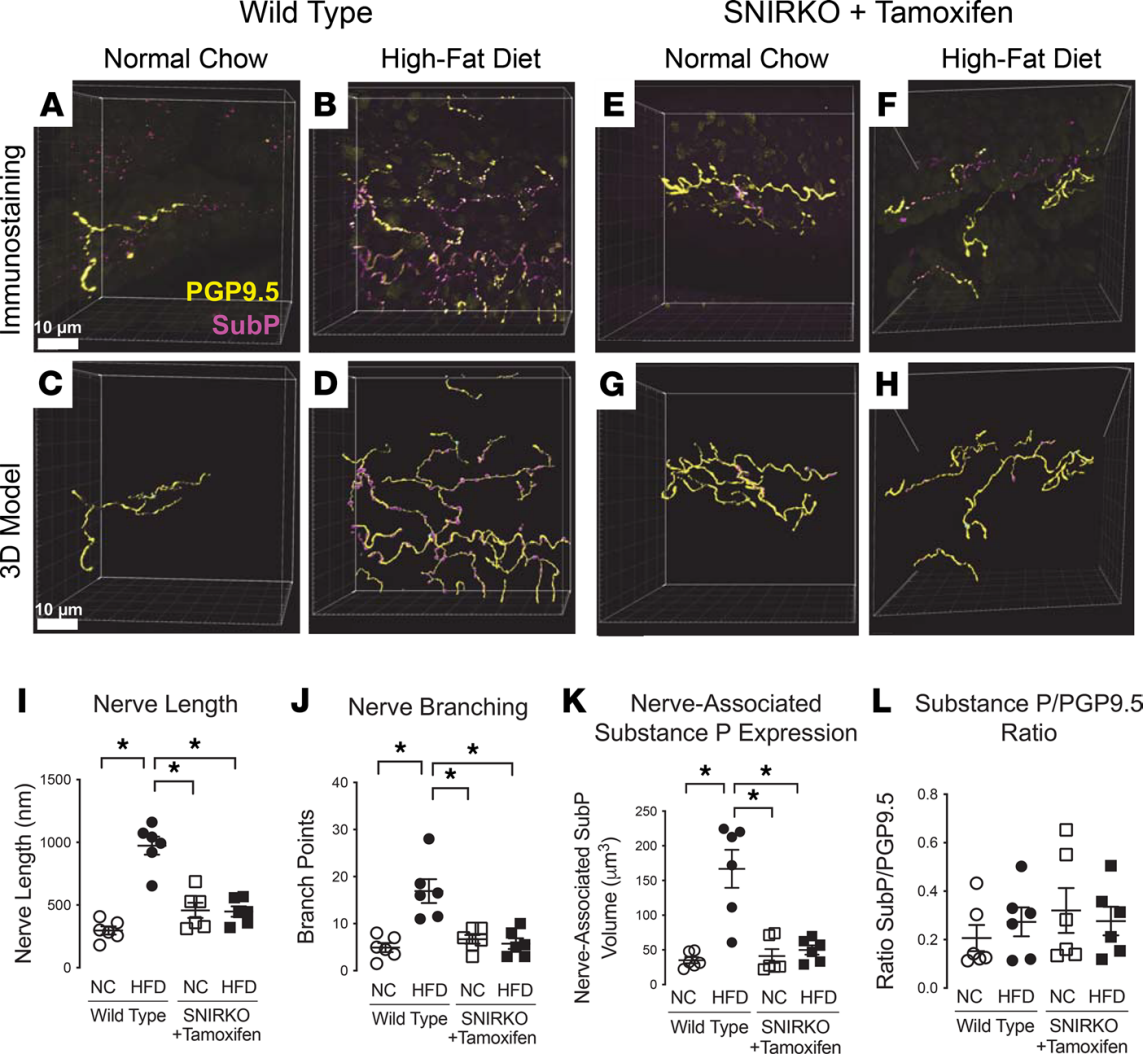
Airway epithelial sensory hyperinnervation induced by a high-fat diet was prevented by decreasing insulin receptors on sensory nerves. (**A**, **B**, **E**, and **F**) Shown are representative images of whole mouse tracheas, labeled with antibody against panneuronal marker PGP9.5 (yellow) and neuropeptide substance P (magenta). Tracheas were optically cleared, imaged using laser scanning microscopy, and imaged as a *Z* stack. (**C**, **D**, **G**, and **H**) Imaris software was used to trace and identify epithelial nerves (yellow), branch points (cyan), and substance p expression (magenta) in WT mice fed normal chow (**C**) or high-fat diet (**D**) and SNIRKO mice fed normal chow (**G**) or high-fat diet (**H**). (**I** and **J**) WT mice on a high-fat diet had increased airway epithelial nerve length (**I**) and number of nerve branch points (**J**). This increase in nerve length, and branching was inhibited when insulin receptors were depleted from sensory nerves of SNIRKO mice. (**K**) High-fat diet–fed mice had increased nerve-associated substance P expression, inhibited by selectively knocking out the insulin receptors. (**L**) All mice had a similar ratio of substance P/PGP. Each data point represents an individual animal; data are represented as mean ± SEM using 1-way ANOVA with Bonferroni post hoc test (*n* = 6). *P < 0.05.

The purpose of this experiment was to determine if changes in airway sensory nerve structure corresponded with increased insulin and airway hyperreactivity. Airway epithelial nerves were imaged using laser scanning microscopy and were modeled on 3D software (Figure 7, A–H). The total length of airway epithelial nerves and total number of nerve branch points were significantly increased in WT mice on a high-fat diet (Figure 7, I and J). The total amount of neuronal substance P expression, determined by colocalization of substance P immunostaining with PGP9.5 expression–positive neurons, was significantly higher in WT mice on a high-fat diet (Figure 7K). A high-fat diet, however, did not lead to an increase in the ratio of neuronal substance P expression to PGP9.5 expression, which indicates that the increase in colocalized substance P was due to an increase in total epithelial nerves, including those that express substance P (Figure 7L). Depleting insulin receptors on sensory nerves in SNIRKO mice on high-fat diet prevented increased airway epithelial nerve length and branching and nerve-associated substance P expression (Figure 7, I–L).

## Discussion

Our data show that diet-induced obese mice have increased circulating insulin, airway epithelial sensory hyperinnervation, and hyperreactivity to both 5-HT and MCh. Hyperreactivity in obese mice was entirely reflex mediated, since vagotomy reduced both 5-HT– and MCh-induced bronchoconstriction to match lean animals. Airway hyperinnervation and hyperreactivity were prevented by selectively depleting insulin receptors on sensory nerves, despite high circulating insulin levels in obese mice, demonstrating that both hyperinnervation and hyperreactivity were due to a direct effect of insulin on sensory nerves. Thus, increased insulin is a key mediator of obesity-related airway hyperreactivity, likely through increased density of sensory nerves expressing substance P. Specifically blocking insulin receptors in sensory nerves prevented obesity-induced hyperinnervation and increased substance P and airway hyperreactivity.

Airway reflex hyperreactivity is determined by airway nerve function, as well as airway smooth muscle contraction, in animals with intact vagus nerves. In vagotomized animals, however, the inhaled MCh-induced bronchoconstriction is mediated entirely by M_3_ muscarinic receptors on smooth muscle. Inhaled MCh acts as a direct agonist of M_3_ muscarinic receptors on airway smooth muscle and acts through activation of airway sensory nerves via a reflex mechanism (25). The response to MCh is potentiated in our prevagotomized obese animals, similar to previous data that come from nonvagotomized mice (22). However, the normalization of this response after vagotomy shows that it actually is the reflex component that is increased, while the direct smooth muscle response is normal. This is consistent with other models of obesity-related airway hyperreactivity (13, 26). Our data also demonstrate that the direct smooth muscle response to inhaled MCh is not altered by selectively depleting the insulin receptor on sensory nerves. Therefore, the normal airway response to 5-HT seen in these mice is not due to a reduction in the contractile response of airway smooth muscle to muscarinic receptor agonists.

This study is the first to demonstrate the importance of airway sensory nerves in obesity-related airway hyperreactivity. Increased airway sensory innervation is present in patients with eosinophilic asthma and in mice that overexpress IL-5 in airway epithelium, causing airway eosinophilia (17). While increased innervation and hyperreactivity in those previous studies were clearly related to inflammation, our current study shows that obesity-induced hyperinnervation and hyperreactivity clearly occur in the absence of airway inflammation and eosinophilia. Although obesity and metabolic syndrome are associated with general systemic inflammation, 4 large clinical studies found no relationship between obesity-associated asthma and the cellular airway inflammation normally associated with allergic asthma (27–30). Studies in diet-induced obese C57BL/6 mice also show increased bronchoconstriction does not rely on inflammatory cell infiltration (31). Lack of airway inflammation in some obesity-related patients with asthma may explain why corticosteroids are less effective in their treatment (32, 33).

Our current study clearly demonstrates a central role for hyperinsulinemia and for a direct effect of insulin on airway sensory nerves in obesity-induced hyperreactivity and hyperinnervation. Recent human birth cohort studies have shown that higher blood insulin in early childhood is associated with a later increased asthma risk and reduced lung function in adulthood, independent of BMI (34). Furthermore, insulin binding to its receptor exerts trophic effects on neurons (21, 35, 36), which could explain the increased airway innervation in diet-induced obese mice. Sensory neurons express insulin receptors and, although their uptake of glucose is not insulin dependent, they respond to insulin signaling (37). Insulin administration has also been shown to prevent sensory nerve atrophy in peripheral diabetic neuropathy (38), indicating an important role of insulin in neuron growth and maintenance.

Advillin expression has been found in most neural crest cell–derived neurons, including some sympathetic and parasympathetic neurons (39). Thus, in SNIRKO mice, tamoxifen-induced Cre may be present in both sympathetic and parasympathetic neurons and deplete insulin receptors in these tissues. Furthermore, the selective depletion of insulin receptors was performed in adult mice in our study, which helped us avoid the early ablation caused developmental defects. Because depleting insulin receptors in SNIRKO mice on a high-fat diet inhibited increased reflex bronchoconstriction, this could also mean that insulin may act on nerves in the autonomic nervous system to cause airway hyperreactivity. Indeed, in obese rats, insulin also inhibits the function of neuronal M_2_ muscarinic receptors on parasympathetic nerves, leading to increased acetylcholine release and increased bronchoconstriction in response to vagal nerve stimulation (13). Combined with the data here, we have shown that insulin affects both the afferent and efferent pathways of the neural reflex, which, combined, cause airway hyperreactivity and are potentially a physiological mechanism for obesity-related asthma.

Potentiation of reflex bronchoconstriction in high-fat diet–induced obese mice could also be mediated by increased airway epithelial nerve responsiveness. In skin, increased airway epithelial nerve responsiveness is associated with increased expression of substance P, which has been shown in inflammatory and pain models (40, 41). In patients with asthma, the number and length of substance P immunoreactive nerves are increased (17). We show that substance P–positive nerves have increased length and branching in hyperinsulinemic, obese mice, consistent with previous reports that substance P–expressing sensory neurons are one of the major subpopulations responding to insulin with neurite outgrowth (42, 43). Since increased substance P was correlated with increased length and branching, our data show that increased substance P expression is likely due to increased density of sensory innervation of the airway.

Normal insulin signaling occurs through 2 major pathways, phosphoinositide-3 kinase (PI3K)/Akt and Ras/mitogen-activated protein kinase (MAPK) pathways (44). One explanation for insulin-induced increased airway innervation is an imbalance in these signaling pathways. Insulin resistance is associated with impairment of the PI3K pathway, while the MAPK pathway often continues to respond to insulin (45, 46). This is important since the MAPK cascade — including extracellular signal-regulated kinases-1 (ERK1), ERK2, and p38 — has an important role in cell growth, survival, and differentiation. ERKs are activated in response to growth factors, including insulin and nerve growth factor (NGF) (47), to induce cell growth and regulate synaptic and structural plasticity in neurons (48, 49). The MAPK-ERK cascade leads to p38 MAPK activation. p38 MAPKs are stress-activated kinases that have been implicated in inflammation (50, 51) and neuronal plasticity (52–54). Therefore, excessive insulin leading to aberrant ERK and p38 activation may contribute to neuronal remodeling in insulin-resistant obese mice and increase sensory innervation of the airways.

Here, we demonstrate an important role for insulin in altering airway sensory innervation, leading to obesity-related airway hyperreactivity. Obese mice have increased nerve-mediated reflex bronchoconstriction and airway sensory nerve hyperinnervation via activation of insulin receptors on sensory nerves. Selective depletion of insulin receptors from sensory nerves prevents increased reflex bronchoconstriction and increased airway innervation in obese mice. As increased density of airway sensory nerves is a feature of human asthma, our findings underscore the previously undefined role insulin plays in mediating obesity-related asthma and further reveal the cellular pathway and mechanism of insulin action. Our results suggest that insulin receptors on specific cells might be considered novel pharmacological targets for obesity-mediated asthma. For patients with asthma and type 2 diabetes, the effect of insulin on asthma control should be carefully considered while controlling blood glucose. For patients with obesity-related asthma, our data indicate that blocking neural control in the lungs may be an effective strategy to prevent or reduce asthma exacerbation.

## Methods

### Animals.

Adult male and female mice (10–30 weeks old) were used for experiments. WT C57BL/6 mice were purchased from The Jackson Laboratory and bred in-house. Transgenic mice with loxp sites flanking exon 4 of the insulin receptor gene (IR^lox/lox^, B6.129S4[FVB]-Insrtm1Khn/J; no. 006955) and mice expressing Cre recombinase driven by mouse sensory nerve specific advillin promoter elements (Avil^icre/+^, Tg[Avil-icre/ERT2]AJwo/J; no. 032027) were purchased from The Jackson Laboratory and bred in-house. Avil^icre/+^ mice were on a mixed C57BL/6 and B6129SF1/J genetic background. Insulin receptor floxed mice were on a 129S4/SvJae background. We bred IR^lox/lox^ with Avil^icre/+^ mice to create creERT2 tamoxifen-inducible SNIRKO mice as described by Grote et al. (24) SNIRKO mice ages 8–10 weeks were treated with 75 mg/kg tamoxifen (20 mg/mL in corn oil, i.p.) for 5 consecutive days to induce Cre expression (Figure 5A). Additional control mice included littermate WT mice given tamoxifen and SNIRKO mice given corn oil (Figure 5A). Although tamoxifen is known to be toxic, short-term tamoxifen treatment has not been shown to interfere with adult neurogenesis (55). Mice were given ad libitum access to food and water and housed on a 12-hour light/dark cycle.

### Diet.

Ten- to 12-week-old male and female mice were given access to either a pelleted high-fat diet (61.6% fat, 18.1% protein, and 20.3% carbohydrate; TestDiet, 58Y1) or a pelleted normal chow diet (13.4% fat, 29.3% protein, and 29.3% carbohydrate; ResearchDiet, 5L0D) for 19 weeks to induce obesity (Figure 1A and Table 1). Mice were housed 2–5 per cage. Food intake for each cage of mice was measured once a week by subtracting the remaining food from the food given. Caloric intake was calculated by multiplying the amount of food consumed by the calorie content per gram of food. The caloric intake data points represent the average number of calories consumed by a single mouse in each cage.

### Body fat, glucose, and insulin measurements.

In order to verify our diet-induced obese mice model, we measured mice’s body fat using a nuclear magnetic resonance–based (NMR-based) body composition analyzer (EchoMRI) as previously described (14). Briefly, mice that were awake were placed into a holding tube and then into a calibrated EchoMRI system. This system uses NMR relaxometry to detect and calculate grams of body water, as well as fat and lean mass, based on the different spin relaxation rates in variable tissues. Blood samples were withdrawn from the inferior vena cava to measure blood glucose (OneTouch Ultra2, LifeScan Inc.) and plasma insulin (mouse insulin ELISA, 10-1247-01, Mercodia). In order to remove the effect of variable circulating insulin on the experiments, mice were fasted for 16 hours before collecting blood and testing airway physiology. Increase in body weight and fat was calculated by taking the difference between diet groups and by dividing by the average of the normal chow control group.

### BALF.

To measure airway inflammation, mice were sacrificed by anesthetic overdose, and leukocytes were harvested from BALF, as previously described (56). Briefly, BALF cells were washed, resuspended in PBS, spun onto slides, and stained with Hemacolor Stain Set (MilliporeSigma, 65044) to obtain a differential cell count.

### Ventilation.

Mice were anesthetized with ketamine (100 mg/kg i.p.) and xylazine (10 mg/kg i.p.), tracheotomized, and ventilated as previously described (56). A 20-gauge catheter was inserted into the cricothyroid membrane to the level of the fourth cartilage ring. Mice were mechanically ventilated with 100% oxygen at 120 breaths/minute with a tidal volume of 200 μL and a positive end-expiratory pressure of 2 cm H_2_O. Mice were paralyzed with succinylcholine (10 mg/kg i.m.) to eliminate respiratory effort. Body temperature was maintained at 37°C with a heat lamp and homeothermic blanket and was measured by rectal probe. Heart rate and rhythm were measured by electrocardiogram recorded with electrodes placed s.c. on the right foreleg, right back shoulder, and left rear leg. Airway pressure, tidal volume, air flow, heart rate, and body temperature were all measured using LabChart Pro acquisition software (ADInstruments).

### Measuring airway resistance.

Airway resistance was measured as previously described (56). Briefly, 2 deep inspirations at 25 cmH_2_O were given, followed by an inspiratory pause for 225 ms at peak inspiration for 4 breaths in a row. For each breath, both peak pressure (P_peak_) and end-inflation pressure (P_plateau_) were recorded, and resistance was calculated as the average (P_peak_ − P_plateau_)/inspiratory flow of these 4 breaths.

Baseline airway resistance was measured at the beginning of each experiment. It was measured again 10 seconds before and 35 seconds after each dose of either aerosolized saline, 5-HT (10 μL, 0–300 mM; MilliporeSigma), or MCh (10 μL, 0–1,000 mM; MilliporeSigma). Change in airway resistance (bronchoconstriction) is graphed as the difference between airway resistance after aerosolized challenge and airway resistance immediately before challenge (cmH_2_O/mL/s). Two deep inspirations were given between each dose to ensure return to baseline between treatments.

### Measuring airway nerve–mediated reflex bronchoconstriction.

Neuronal contribution to bronchoconstriction was measured by comparing changes in airway resistance in response to inhaled 5-HT (10 μL, 10–300 mM) with and without vagotomy, as previous described (56). Vagotomy and atropine (3 mg/kg i.p.) were used to test whether responses were vagally mediated. In some animals, during ventilation experiments, 2 dose-response curves to 5-HT were recorded — the first with the vagi intact and the second after both vagus nerves were isolated and cut with microdissection scissors to eliminate reflex (neuronal) bronchoconstriction (bilateral vagotomy). In control animals, bilateral cervical vagus nerves were isolated but not cut. In other animals, after a first dose-response curve, atropine was given and the response to the highest dose of 5-HT was again tested.

### Measuring M_3_ muscarinic receptor function.

The function of M_3_ muscarinic receptors on airway smooth muscle was measured using the change in airway resistance in response to increasing doses of aerosolized MCh (10 μL, 10–300 mM) in animals that were vagotomized to eliminate the reflex component of MCh-induced bronchoconstriction (25, 57).

### Tissue optical clearing and imaging, and quantification of airway nerves.

Mice were perfused with phosphate buffered saline (PBS), and airways were excised. Tracheas were left at 4°C in Zamboni fixative (Newcomer Supply) overnight. Tracheas were blocked overnight with 4% normal goat serum, 1% Triton X-100, and 5% powdered milk; they were then incubated with antibodies to pan-neuronal marker protein gene product 9.5 (PGP9.5, rabbit IgG, 1:200; Amsbio) and substance P (rat IgG_2A_, 1:500; BD Pharmingen) on a shaker at 4°C overnight. Tissues were washed and incubated overnight in secondary antibodies: Alexa Fluor goat anti–rabbit 647 (1:1,000; Invitrogen) and Alexa Fluor goat anti–rat 555 (1:1,000; Invitrogen) and counterstained using the nuclear stain DAPI (Molecular Probes). Tracheas were then optically cleared in N-methylacetamide/Histodenz (Ce3D) for 12 hours (58) and mounted in Ce3D on slides in 120 μm–deep imaging wells (Invitrogen).

Images were acquired using a Zeiss LSM900 confocal microscope and 63×/1.4 oil PlanApo DIC M27 objective with a 0.19 mm working distance. Samples were illuminated with 401 nm, 553 nm, and 653 nm light, and images were acquired as *Z* stacks. Two to 3 randomized images were taken of each mouse trachea using DAPI to locate the epithelium. Airway nerves were modeled in 3D using Imaris semiautomatic filament tracing software (Imaris 9.7, Oxford Instruments). Users were blinded to study group at the time of nerve modeling. A 3D filament model was created around tracheal epithelial nerves to quantify nerve length and number of branch points. Neuronal substance P expression was quantified by creating a surface around the substance P–positive voxels and using Imaris software to colocalize the voxels in contact with PGP9.5^+^ nerve axons. Two to 3 images were quantified per mouse trachea.

### Confirmation of insulin receptor KO using qPCR.

Depletion of the insulin receptors on sensory nerves was confirmed via qPCR. Mice were euthanized with anesthetic overdose and perfused with PBS, and tissue was dissected and snap frozen in liquid nitrogen. Total RNA was isolated from control and SNIRKO liver, nodose and jugular ganglia, and DRG using the RNeasy Mini Kit (Qiagen). cDNA was generated using Superscript III Reverse Transcriptase (Thermo Fisher Scientific). Insulin receptor mRNA expression was quantified using QuantiTect SYBR Green (Qiagen) and qPCR (7500 Fast Real-Time PCR System, Applied Biosystems). Insulin receptor primers were: left primer 5′-GAGGCTGCACTGTGATCAAC-3′ and right primer (located within exon 4) 5′-TAGGAGCGGCGGATCTTTAG-3′. Primers for the housekeeping gene 18S rRNA were 5′-GTAACCCGTTGAACCCCATT-3′ and 18S 5′-CCATCCAATCGGTAGTAGCG-3′. Insulin receptor expression data were analyzed using the ΔΔCT method (59) and normalized to 18S rRNA.

### Insulin receptor mRNA localization using BaseScope.

To measure whether insulin receptors were selectively depleted in sensory tissue, BaseScope (Advanced Cell Diagnostics) mRNA visualization was used. Tissues were fixed in 10% neutral buffered formalin overnight at room temperature. BaseScope assays were performed on paraffin sections (5 μm thickness Superfrost plus slides, Thermo Fisher Scientific) using guidelines provided by the supplier (Advanced Cell Diagnostics). Briefly, slides were baked at 60°C for 1 hour before deparaffinizing in xylene (2 × 5 min) and ethanol (2 × 2 min); they were then dried at 60°C for 5 minutes. RNAScope hydrogen peroxide was applied for 10 minutes at room temperature, then target retrieval was applied for 15 minutes at 100°C. RNAScope protease III was then applied for 30 minutes at 40°C. BaseScope insulin receptor (catalog 719141), positive control (catalog 01071), and negative control probes (catalog 701011) were purchased from Advanced Cell Diagnostics and in situ hybridized to tissue sections with BaseScope Detection Reagent Kit v2 - RED (Advanced Cell Diagnostics). Slides were counterstained with 50% Gill’s hematoxylin and then 0.02% ammonia water before drying for 15 minutes at 60°C and mounting in EcoMount (Biocare Medical).

### Statistics.

Increases in airway resistance in response to inhaled nebulized MCh and 5-HT data were analyzed using 2-way repeated-measures ANOVA (60). Body weight and fat, caloric intake, fasting blood glucose, and insulin were analyzed by 2-tailed Student’s *t* test and 1-way ANOVA. Nerve length, branching, and substance P data were analyzed by 1-way ANOVA with Bonferroni post hoc test. All data were analyzed with Prism 8.0 software (GraphPad). *P* < 0.05 was considered significant.

### Study approval.

Animals were handled in accordance with standards established by the *Guide for the Care and Use of Laboratory Animals* (National Academies Press, 2011) and approved by the IACUC at Oregon Health & Science University.

## Author contributions

GNC, JNM, and ZN carried out the experiments. GNC and ZN analyzed data and wrote the manuscript. ZN, DBJ, and ADF participated in the experimental design and the revision of the manuscript.

## Supplementary Material

Supplemental data

## Figures and Tables

**Table 1:**
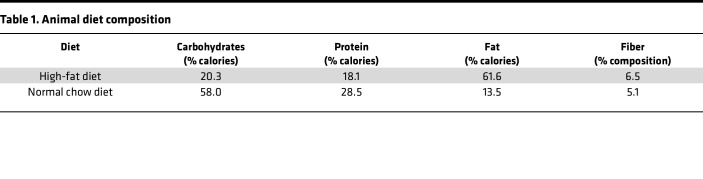
Animal diet composition

## Version 1 09/15/2022

In-Press Preview

## Version 2 10/24/2022

Electronic publication
