# Supplementary material for: Insulin increases sensory nerve density and reflex bronchoconstriction in obese mice
Source: JCI Insight. 2022 Oct 24;7(20):e161898. doi: 10.1172/jci.insight.161898 (PMC9714782; doi:10.1172/jci.insight.161898)
Supplement: Supplemental data [file jciinsight-7-161898-s024.pdf]

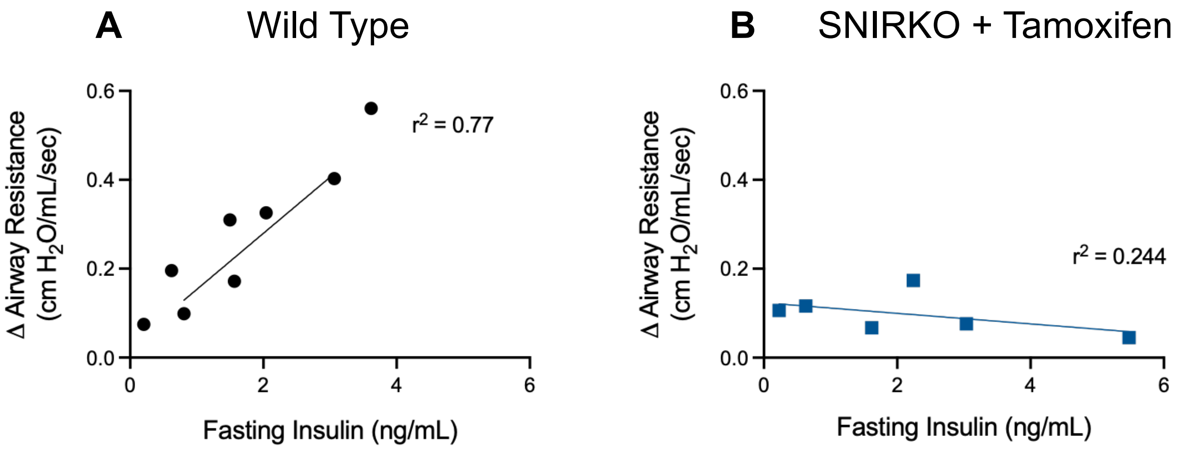

Supplemental Figure 1. Change in airway resistance increased with fasting insulin in wild type but not SNIRKO mice. Airway resistance in response to 300 mM 5-HT directly correlates with fasting insulin in wild type (A) but not SNIRKO (B) mice. Each data point represents an individual animal. (n = 6-8). Simple linear regression.
